# Community diversity and the other-race effect in infancy

**DOI:** 10.3389/fpsyg.2023.1214075

**Published:** 2023-09-11

**Authors:** Trinity Bauer, Cidnee Hall, Aslı Bursalıoğlu, Maggie W. Guy

**Affiliations:** Department of Psychology, Loyola University Chicago, Chicago, IL, United States

**Keywords:** other-race effect, infancy, face recognition, visual attention, racial diversity

## Abstract

The other-race effect (ORE) is characterized by processing advantages for faces of one's own race over faces of another race and is observed at ~9 months of age. Environmental exposure to other races has an impact on the development of the ORE. In the current study, we examined the effects of community racial diversity on the ORE in 9- to 12-month-olds from across the United States. We hypothesized that community racial diversity would influence the amount of experience that infants have with individuals of other races and be an important factor in predicting the ORE across broad regions of the United States. We predicted that infants from more diverse communities would demonstrate successful processing of own- and other-race faces, while infants from less diverse communities would demonstrate successful processing of own-race but not other-race faces. This would indicate that the ORE is exhibited more strongly in infants from less diverse communities than in infants from more diverse communities. Participants completed familiarization and visual paired comparison (VPC) trials with own- and other-race faces in an online study. Our results showed that although the ORE was present, the effect was driven by community members who were the racial majority. Recognition biases were not observed in community racial or ethnic minority participants, potentially due to increased exposure to racial out-group members, which mitigated the development of the ORE in this subset of participants. This study has far-reaching implications in the study of infant face perception, child development, and social justice, as the ORE develops at a young age, and may lead to a complex pattern of racial biases contributing to systemic barriers in society.

## Introduction

The formation of social categories is a foundational component of human interaction that guides social learning, cognition, and behavior in children and adults (e.g., Quinn et al., 2019; Rhodes and Baron, 2019). One aspect of social categorization is the development of in- and out-groups, which contribute to the development of prejudice and stereotypes (Rhodes and Baron, 2019). Given the importance of in- and out-group categorization in driving social behavior, understanding the processes by which infants initially develop social categories could inform our understanding of the basis of implicit biases in adulthood (Rhodes and Baron, 2019; Quinn et al., 2020). Racial categories are a particularly important marker of social identity, and both the pervasiveness and stability of implicit racial biases among adults in the United States reflect a need to understand the development of implicit bias as well as ways to attenuate its negative impact (Nosek et al., 2007; Lai et al., 2016). The other-race effect (ORE) is one possible mechanism contributing to the development of implicit racial bias (Lee et al., 2017; Quinn et al., 2018, 2019). The ORE is a perceptual phenomenon whereby individuals process faces of other races less efficiently and have poorer recognition of other-race faces compared to faces of their own racial identity (Kelly et al., 2007; Rhodes and Baron, 2019; Serafini and Pesciarelli, 2022). The ORE emerges in infancy, but the strength of the effect may depend on early individual experience with faces of diverse races and ethnicities (Sangrigoli and De Schonen, 2004; Sangrigoli et al., 2005; Bar-Haim et al., 2006; Heron-Delaney et al., 2011; Spangler et al., 2013; Sugden, 2016; Tham et al., 2019; Zhou et al., 2019, 2022; Hwang et al., 2021). The current study aimed to examine whether community diversity, as measured by zip code, relates to the ORE exhibited by 9- to 12-month-old infants across the United States.

The ORE may result from perceptual narrowing in infancy. Perceptual narrowing is a developmental process by which visual discrimination capabilities become more “fine-tuned” to frequently encountered stimuli across the first year of life (Kelly et al., 2007, 2009; Scott et al., 2007; Pascalis et al., 2020). Many mechanisms have been proposed to underlie perceptual narrowing for own-race faces, including familiarity, experience-dependent socio-cultural factors, and the development of category representations (Quinn et al., 2016; Markant and Scott, 2018; Marquis and Sugden, 2019; Pascalis et al., 2020; Damon et al., 2022). There does appear to be an innate component for increased visual attention to faces, as evidenced by newborns who, within a few hours from birth, exhibit a visual preference for faces and face-like stimuli over other types of stimuli (Goren et al., 1975; Johnson et al., 1991). Experiences begin shaping these preferences as early as 3 months of age when infants have demonstrated increased attention toward faces of their own race in preferential-looking paradigms (Kelly et al., 2005; Marquis and Sugden, 2019). Because infants are primarily exposed to the face of their primary caregiver, who is most often an adult female of their own race, this archetype may form the basis by which narrowing recognition abilities and visual preference for age, gender, and race occur (Macchi Cassia et al., 2014; Sugden and Moulson, 2019; Bayet, 2022). Evidence from these preferential-looking paradigms may be explained by the emergence of advantaged face processing for own-race faces (Liu et al., 2015; Fassbender et al., 2016; Quinn et al., 2019).

The ORE develops after these perceptual preferences emerge, at approximately 9 months of age (Sangrigoli and De Schonen, 2004; Kelly et al., 2007, 2009; Quinn et al., 2018, 2019; Krasotkina et al., 2020; Pascalis et al., 2020). Foundational studies in the development of the ORE in infancy (e.g., Sangrigoli and De Schonen, 2004; Kelly et al., 2007, 2009) have utilized a paradigm in which infants are familiarized or habituated with a stimulus and then tested for recognition of this stimulus using a visual paired comparison (VPC) including side-by-side presentations of the familiarized stimulus and a novel stimulus (Fantz, 1956; Fagan, 1970). A novelty preference is seen when a greater proportion of time is spent looking at the novel stimulus, which indicates the recognition of the familiar stimulus (Fantz, 1956; Fagan, 1970; Richmond et al., 2007). Sangrigoli and De Schonen (2004) used this paradigm with a sample of White infants in the first study to examine the ORE in infancy. Three-month-old participants were habituated to a face and then viewed a familiar face beside a novel face in a VPC trial. For one set of trials, participants viewed White faces, and for another set of trials, participants viewed Asian faces. The researchers found that infants were more likely to demonstrate a novelty preference on the own-race (i.e., White) trials, indicating the recognition of the habituated own-race face. This was not seen on other-race faces.

This procedure has been used in numerous infant ORE studies, including foundational studies by Kelly et al. (2007, 2009) that explored the development of the ORE across the first year of life. In total, 3-, 6-, and 9-month-old White infants' ability to process faces of Black, Asian, Middle Eastern, and White races was tested using a VPC task (Kelly et al., 2007). They found that infants demonstrated the recognition of familiar faces of all races at 3 months, recognition for both Asian and White faces at 6 months, and recognition only for White faces at 9 months (Kelly et al., 2007). Kelly et al. (2009) obtained very similar results using a VPC task to examine 3-, 6-, and 9-month-old Chinese infants' responses to Black, White, and Chinese faces, with infants displaying the recognition of all faces at 3 months, White and Chinese faces at 6 months, and only Chinese faces at 9 months.

Additional research has shown that the presence and magnitude of the ORE are sensitive to experience. Even brief experiences with other-race faces, including short sessions of exposure to other-race faces and perceptual training for other-race faces through picture books, have been shown to attenuate the ORE in infants who were primarily exposed to faces of their own race in their daily lives (Sangrigoli and De Schonen, 2004; Heron-Delaney et al., 2011; Spangler et al., 2013). Language experience may additionally influence the ORE. Recent evidence has shown that bilingual language exposure reduces the strength of the ORE (Burns et al., 2019). Furthermore, exposure to static pictures of faces paired with soundtracks containing participants' native and non-native languages has influenced the presentation of ORE in infancy (Hillairet de Boisferon et al., 2021; Ujiie et al., 2021; Clerc et al., 2022).

Several studies have looked at the impact of exposure to racial diversity in naturalistic settings on the development of the other-race effect and found promising results toward changing the developmental course of the ORE (Sangrigoli et al., 2005; Bar-Haim et al., 2006; Sugden, 2016; Tham et al., 2019; Zhou et al., 2019, 2022; Hwang et al., 2021). Studies of adults have been particularly informative in this area, using lifetime experience in diverse communities as a predictor of own-race biases. In a recognition memory task of Asian and White faces, Sangrigoli et al. (2005) demonstrated that Korean participants adopted by White Europeans as infants performed similarly to White European participants with greater recognition of White faces compared to Asian faces, in contrast to Korean participants living in Korea who had a greater recognition of Asian faces. Similar results have been reported in studies of White and East Asian participants in Canada, finding that White Canadians and East Asian adults who immigrated to Canada showed the ORE, whereas East Asian adults born and raised in Canada did not (Zhou et al., 2019). The age of immigration to the country was positively correlated with the strength of the ORE, further emphasizing the impact of early experience on shaping later face recognition skills (Zhou et al., 2022). Even within cities, the relative diversity of the community one lives in may impact the magnitude of the ORE in adult participants (Zhou et al., 2022).

Studies of exposure to diversity have also been conducted with infant participants. Bar-Haim et al. (2006) sampled Black infants raised in Ethiopia, White infants raised in Israel, and Black infants raised in Israel to parse out the unique effects of being a racial majority or minority member on the development of visual attention preferences for own- and other-race faces. Although the authors found that a majority of members (Ethiopian and Israeli in their native countries) exhibited a preference for own-race faces as early as 3 months of age, surprisingly, the Black infants raised in Israel did not. Bar-Haim et al.'s (2006) results indicate that although the ORE is observed cross-culturally, it may not be uniform across individuals with varying racial identities within a specific location. In a similar study, Sugden (2016) examined the other-race effect in a diverse sample of Black, White, East Asian, and biracial infants living in a diverse, metropolitan area in Canada and found no race preference, as well as no discrimination or recognition advantages for any race, in the infants at 3, 6, or 9 months of age. Greater nuance to this effect is described by Tham et al. (2019), who found that Chinese infants from a multi-racial environment in Malaysia were able to recognize their own- and other-race faces with which they had frequent experience (e.g., Chinese and Malay) but were not able to discriminate among faces of other races that were not well-represented in the environment (e.g., White). These findings indicate that the development and strength of the ORE may depend primarily on experience with individuals of other races and ultimately emphasize the importance of examining racial homogeneity and exposure to diversity at the community level to understand the development of the ORE.

Importantly, none of these studies were carried out in the United States, a very racially diverse nation in which people may encounter exemplars of other-race faces regularly depending on the community that they live in. Hwang et al. (2021) attempted to address this question by measuring zip code level diversity in the United States as a modulator of the responses to own- and other-race faces in racial majority and minority infants, finding only White infants' neural responses to people of different races were related to neighborhood diversity. Their results indicate exposure to greater levels of diversity in racial majority infants may slow or prevent the development of perceptual biases for own-race faces (Hwang et al., 2021). However, the study is limited by its use of only two communities, Chicago, IL and College Park, MD, to represent a diverse and monoracial community and by including only three race exemplars (White, East Asian, and South Asian) in the stimuli viewed by the infant participants.

Overall, the literature suggests that infants who are primarily exposed to faces of their own race in their environment experience perceptual narrowing across the first year of life, resulting in more optimized processing and greater recognition of own-race faces compared to those of other races. However, the current body of literature must be considered in the context of several major limitations. General limitations of infant research impact the current scarcity of literature in this area, including difficulty accessing infant populations and high rates of data loss. Both behavioral and neural studies of the ORE additionally lack sample diversity, particularly for Black, Hispanic/Latino, or Indigenous populations (Serafini and Pesciarelli, 2022). Of the infant ORE studies reviewed here, 12 included homogenous samples of White infants (Sangrigoli and De Schonen, 2004; Kelly et al., 2005, 2007; Heron-Delaney et al., 2011; Spangler et al., 2013; Macchi Cassia et al., 2014; Fassbender et al., 2016; Quinn et al., 2016; Krasotkina et al., 2020; Hillairet de Boisferon et al., 2021; Clerc et al., 2022; Damon et al., 2022), four included homogenous samples of East Asian infants (Kelly et al., 2009; Liu et al., 2015; Tham et al., 2019; Ujiie et al., 2021), and only three included any Black, Hispanic, or bi-/multi-racial infants (Bar-Haim et al., 2006; Sugden, 2016; Hwang et al., 2021). The brief review included here is further supported by a recent meta-analysis, which found that over 60% of samples in the infant face discrimination literature were conducted using a homogenous sample of White infants, and another ~25% were conducted using a homogenous sample of East Asian infants (Sugden and Marquis, 2017). These limitations greatly reduce the overall generalizability of the literature. The lack of racial and ethnic diversity in many of these studies reflects that the studies have been carried out in highly racially homogenous countries where infants are expected to have very little other-race exposure or that other-race exposure has been specifically controlled for by recruiting infants who are reported by caregivers to have little to no other-race exposure. Given the findings that living in multi-racial environments may diminish the strength of the ORE, greater investigation into the role of community-level exposure to diversity on the strength of the ORE in infancy is needed to better understand how early social environments shape face processing skills.

The current study aimed to examine the ORE in 9- to 12-month-old infants in relation to community demographics, as measured by participant zip code and data taken from the U.S. Census' American Community Survey (U.S. Census Bureau, 2022). The current study addresses previous limitations of the literature by recruiting participants from a diverse range of communities across the United States via Lookit, an online platform where parents and infants may participate in studies asynchronously (Scott and Schulz, 2017; Scott et al., 2017). The Lookit platform enabled the recruitment of participants from areas of the United States with varying levels of racial and ethnic diversity and allowed us to vary the presentation of stimuli for different combinations of own- and other-race faces in a VPC task. Moreover, the current study did not limit participant recruitment to one racial category, thus allowing us to recruit a sample that more closely reflects the racial and ethnic makeup of the United States. We additionally measured parent self-reports of infant exposure to other-race faces to corroborate our measure of community diversity. Because of our focus on the ORE in relation to community diversity, we focused on infants at 9–12 months of age, when the ORE was already expected to have developed (e.g., Kelly et al., 2007, 2009). We hypothesized that infants living in more diverse communities would exhibit less of a perceptual bias for own-race faces compared with infants living in more homogenous communities, as evidenced by the proportion of looking at own- and other-race faces after familiarization in a VPC task.

## Methods and materials

### Participants

**Figure 1:**
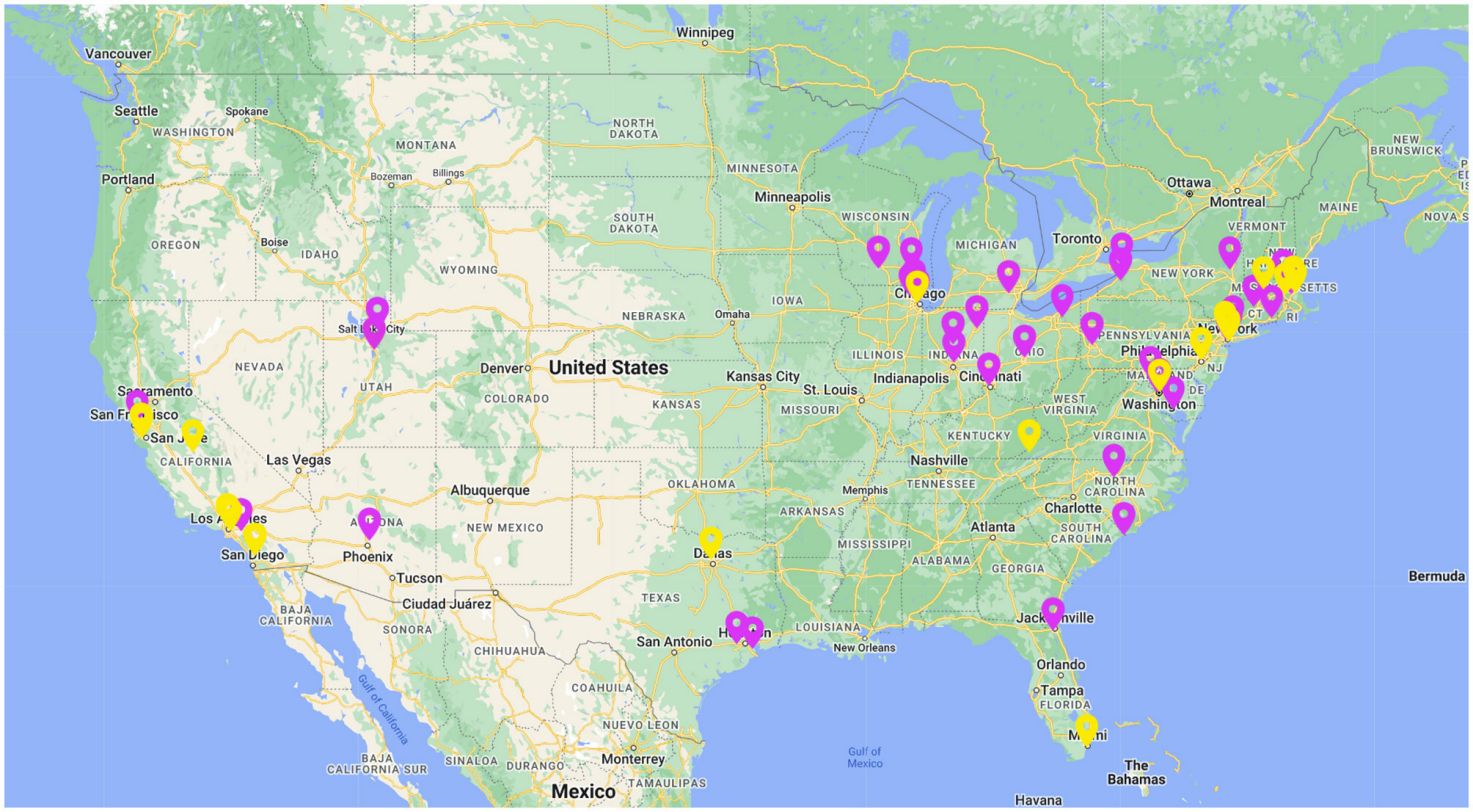
Map of participant zip codes. Yellow represents demographic minority participants, and purple represents demographic majority participants.

A total of 108 infants were recruited for the current study, and a final sample of 67 was retained for analysis. Thirty-eight participants were excluded due to failure to meet looking time criteria (*n* = 16), technical difficulties (*n* = 12), incomplete data (*n* = 11), and inability to match stimuli to a participant's racial identity (*n* = 2). Participants were infants aged 9–12 months (*M* = 322 days; range = 273–396 days) at the time of participation. All participants were typically developing, full-term infants living in the United States. Participant gender, race, and ethnicity demographics are reported in Table 1. Participant zip codes are presented in Figure 1. The study was run, and participants were recruited through the online child development platform Lookit, developed by MIT (Scott and Schulz, 2017; Scott et al., 2017). Studies are published on the Lookit homepage for parents to seek out for participation, or parents registered on the website are emailed by the platform to notify them of a study that their child may be eligible for. Infants' caregivers were compensated with $5 e-gift cards for volunteering to participate.

**Table 1:** Participant demographics.

		***n***	**%**
Gender	Male	36	53.7
	Female	31	46.3
Race	Black	2	2.9
	White	48	71.6
	Asian	6	8.9
	2 or more races	10	20.8
	Not reported	1	1.5
Ethnicity	Hispanic/Latinx	3	4.4
	Not Hispanic/Latinx	52	77.6
	Not reported	10	14.9

### Stimuli

**Figure 2:**
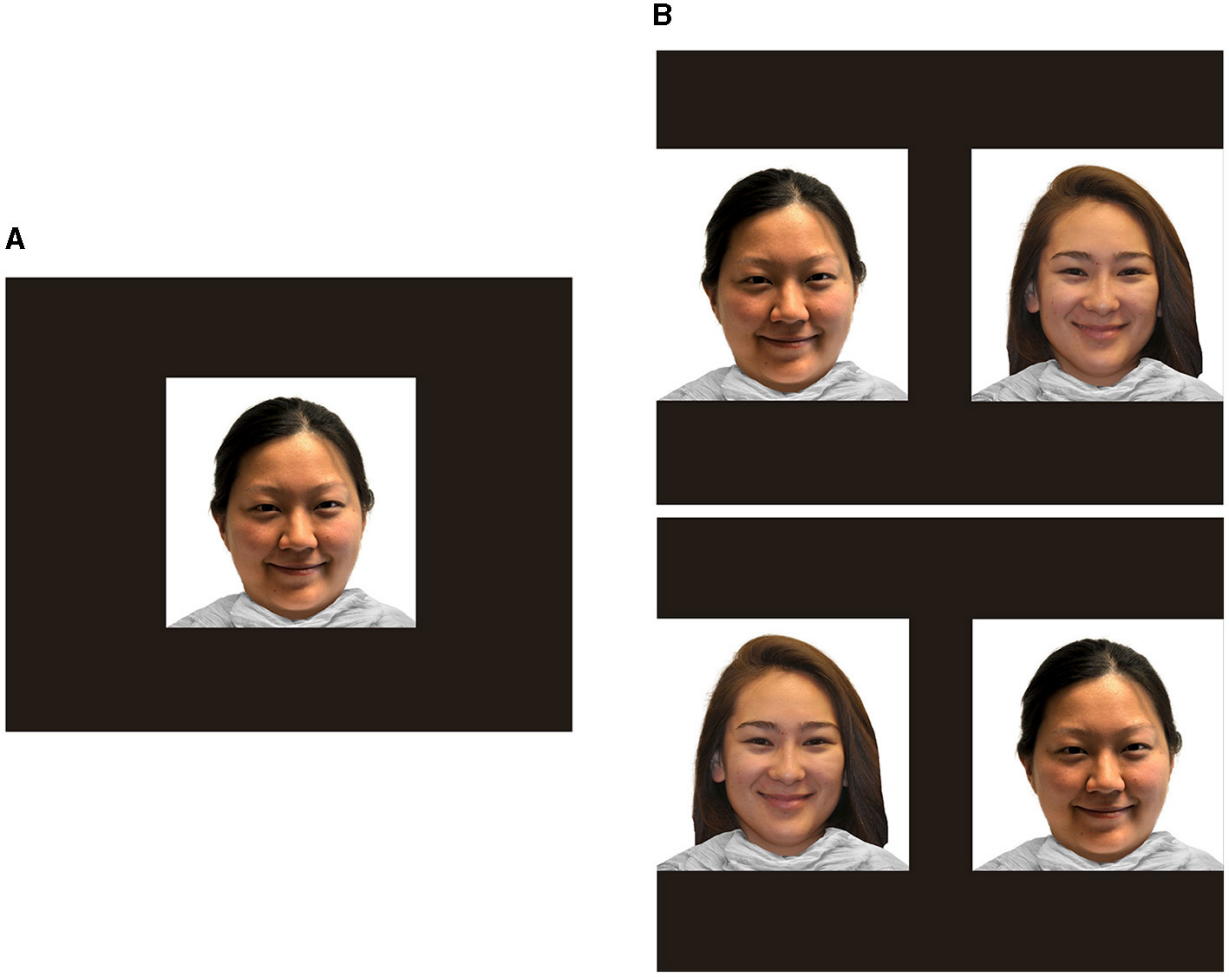
Sample familiarization and visual paired comparison stimuli. **(A)** Presents a sample familiarization stimulus. A single own- or other-race face was presented in the center of the screen for 30 s. **(B)** Shows sample visual paired comparison trial. The familiar face was presented beside a novel face for 10 s. This was repeated with the positions of the faces switched. Facial images reproduced with permission from the Racially Diverse Affective Expression (RADIATE) face stimulus set (Tottenham et al., 2009; Conley et al., 2018).

The stimuli selected were six exemplars for each racial category comprising White women, Black women, Asian women, and Hispanic women from the Racially Diverse Affective Expression (RADIATE) face stimulus set (Tottenham et al., 2009; Conley et al., 2018). All images were forward-facing, color photographs of women wearing white shirts against a white background with a closed-mouth, happy facial expression. Only female faces were used to control for infant-looking biases based on gender, as has been observed in previous research (Quinn et al., 2002, 2008). Although six face exemplars were selected for each racial category because of the uneven demographic distribution of participants, six White female faces, four Black female faces, four Asian female faces, and four Hispanic female faces were utilized from the chosen stimuli. Sample stimuli are presented in Figure 2.

### Procedure

Participants completed all study activities asynchronously online. The university IRB approved all procedures, and parents gave their consent via recorded videos. After completing the informed consent statement, parents were asked to complete a demographic form. The demographic form requested information including their zip code, the participant's racial background, the infant's ethnicity, the race(s) of individuals that the infant is exposed to and interacts with regularly, and the infant's gender. Parents additionally had the option to preview the experimental procedure prior to beginning the experiment. Parents were then instructed to turn their backs to the computer screen and to hold the infant comfortably, looking over their shoulder to face the screen. An attention grabber with audiovisual stimuli was shown to direct the infant's attention to the screen and to provide a reference of the infant's looking at the right, left, and center prior to the presentation of study stimuli. The infant was then shown two sets of familiarization and visual paired comparison trials. Infants completed one trial consisting of own-race familiarization and VPCs, and one trial consisting of other-race familiarization and VPCs. The order of own- or other-race trials was counterbalanced across participants.

#### Familiarization phase

During the familiarization phase, a single face was presented on-screen for 30 s belonging to one of the race categories: White, Black, Asian, or Hispanic. For the own-race familiarizations, all participants were shown a face that most closely matched their specified racial identity. The face stimuli directly matched the parent's selection of their infant's racial identity in most cases (*N* = 57). For multi-racial participants (*N* = 10), infants were shown face stimuli with which they identified with one of the racial backgrounds during the own-race familiarization (e.g., biracial participants who identified as White and Asian were shown Asian faces as own-race stimuli). For the other-race familiarization, all participants were shown an other-race category that did not correspond with any of their selected racial identities.

#### Visual paired comparison trials

During the VPC trials, the infants were presented with the same face from familiarization on one side of the screen, and a novel face of the same race on the other side of the screen. The stimuli were presented for 10 s. Participants were also presented with the same stimuli but on opposite sides of the screen to account for potential side bias, which was not observed in any of the participants.

### Data processing

The data were coded using Datavyu, a software designed for coding developmental behavioral data (Datavyu Team, 2014). Six videos were coded for each participant: one own-race familiarization, one other-race familiarization, two own-race paired comparisons, and two other-race paired comparisons. Videos were coded frame-by-frame by trained raters who were blind to the positions of the novel/familiar faces at the time of coding. Familiarizations were coded for time spent looking at the monitor and time spent looking away from the monitor. The VPC trials were coded for infants' time spent looking at the left side of the monitor, to the right side of the monitor, and away from the monitor. Twenty percent of the data were coded by a second rater to calculate inter-rater reliability, which was high (Pearson's *r* > 0.97). For inclusion in the final data set, participants were required to attend the familiarization stimulus for at least 10 s and the stimuli presented on the VPC trials for at least 3.5 s.

### Measurement of racial diversity

We used the U.S. Census' American Community Survey (U.S. Census Bureau, 2022) and the zip codes provided by parents to retrieve information about community-level racial and ethnic diversity in the communities where participants lived. The Herfindahl–Hirschman index (HHI; Hirschman, 1964; Rhoades, 1993) was further used to calculate a neighborhood diversity score. The Hirschman–Herfindahl index is an economic measure of market concentration and competition and has been applied in previous studies as a measure of community racial and ethnic diversity (Sturgis et al., 2014; Awaworyi Churchill et al., 2019).

The Hirschman–Herfindahl index is represented by:


(1) $$\begin{array}{l} HHI=1-\overset{n}{\underset{i=1}{\sum }}s_{i}^{2} \end{array}$$

where s_i_ is the percentage share of a racial or ethnic group in a given community. The resulting HHI can be interpreted as the probability that two individuals from the same community, when randomly selected, would be of the same racial/ethnic identity. Higher scores on the HHI represent more diverse populations, with scores ranging from 0 (all individuals in the community are of the same race) to 1 (all individuals in the community are of different races). For example, the 60626 zip code, which roughly corresponds to the highly diverse Rogers Park community of Chicago, IL, has an HHI of 0.69, while the 14052 zip code, which roughly corresponds to the highly racially homogenous city of East Aurora, NY, has an HHI of 0.08.

### Analyses

Analyses were conducted in IBM SPSS. Paired samples *t*-tests were used to examine overall differences in looking at own-race familiar and novel faces, as well as overall differences in looking at other-race familiar and novel faces. One-way ANOVAs were conducted to compare the effects of demographic majority status on the proportion of looking at own-race novel and other-race novel faces. A linear regression model was analyzed using neighborhood diversity score as a continuous predictor and proportion of looking at own- and other-race faces as a continuous outcome. The majority- or minority-race status of the infant was additionally included in the model as a dichotomous moderator to examine whether the interaction between diversity and demographic majority status significantly predicted the proportion of looking at own- or other-race novel faces.

## Results

### Descriptive statistics

Participants looked for an average of 19.92 s (min = 11.57 s, max = 29.13 s, *SD* = 4.44 s) during own-race familiarization and 19.73 s (min = 10.84 s, max = 29.95 s, *SD* = 5.32 s) during other-race familiarization. Infants did not differ in their attentiveness to their own- or other-race familiarizations, *t*(61) = 0.41, *p* = 0.680. The total looking time during own-race VPC trials was an average of 8.48 s (min = 3.63 s, max = 12.51 s, *SD* = 1.84 s) and 7.72 s (min = 3.56 s, max = 10.19 s, *SD* = 1.93 s) during other-race VPCs. Infants attended significantly longer own-race VPC trials compared with other-race VPC trials, *t*(61) = 3.04, *p* = 0.003.

Community diversity varied widely among participants (M_HHI_ = 0.47, min = 0.07, max = 0.74, *SD* = 0.18). Parent-reported exposure to own- and other-race individuals also varied among participants. The results of this survey item are presented in Table 2. Bivariate correlations showed that demographic minority status was marginally significantly correlated with diversity index score, such that participants with a minority identity tended to live in more diverse communities, *r*(64) = 0.23, *p* = 0.061. However, the parent that reported exposure to individuals of other races was uncorrelated with both demographic minority status, *r*(48) = −0.09, *p* = 0.537, and diversity index score, *r*(64) = 0.04, *p* = 0.785.

**Table 2:** Reported exposure to own- and other-race individuals.

	***n***	**%**
Own-race exposure only	30 (10)	44.7
Own- and other-race exposure	21 (5)	31.3
Not reported	16 (4)	23.8

Numbers in parentheses represent the number of respondents with racial or ethnic minority identity.

### Test of the other-race effect

In the own-race stimulus condition, paired sample *t*-tests revealed significant differences in looking at own-race familiar and own-race novel faces, *t*(64) = −2.96, *p* = 0.002. Participants looked significantly longer at novel own-race faces (*M* = 4.65 s, *SD* = 1.58 s) than at familiar own-race faces (*M* = 3.83 s, *SD* = 1.29 s). In the other-race stimulus condition, there were no significant differences in looking at familiar and novel other-race faces, *t*(65) = −1.22, *p* = 0.114. Participants did not significantly differ in their looking at novel other-race faces (*M* = 4.06 s, *SD* = 1.71 s) vs. familiar other-race faces (*M* = 3.67 s, *SD* = 1.51 s). These results indicate that participants in the study demonstrated the ORE by showing an advantage for processing own-race, compared with other-race, faces.

### Local community majority status and the ORE

**Figure 3:**
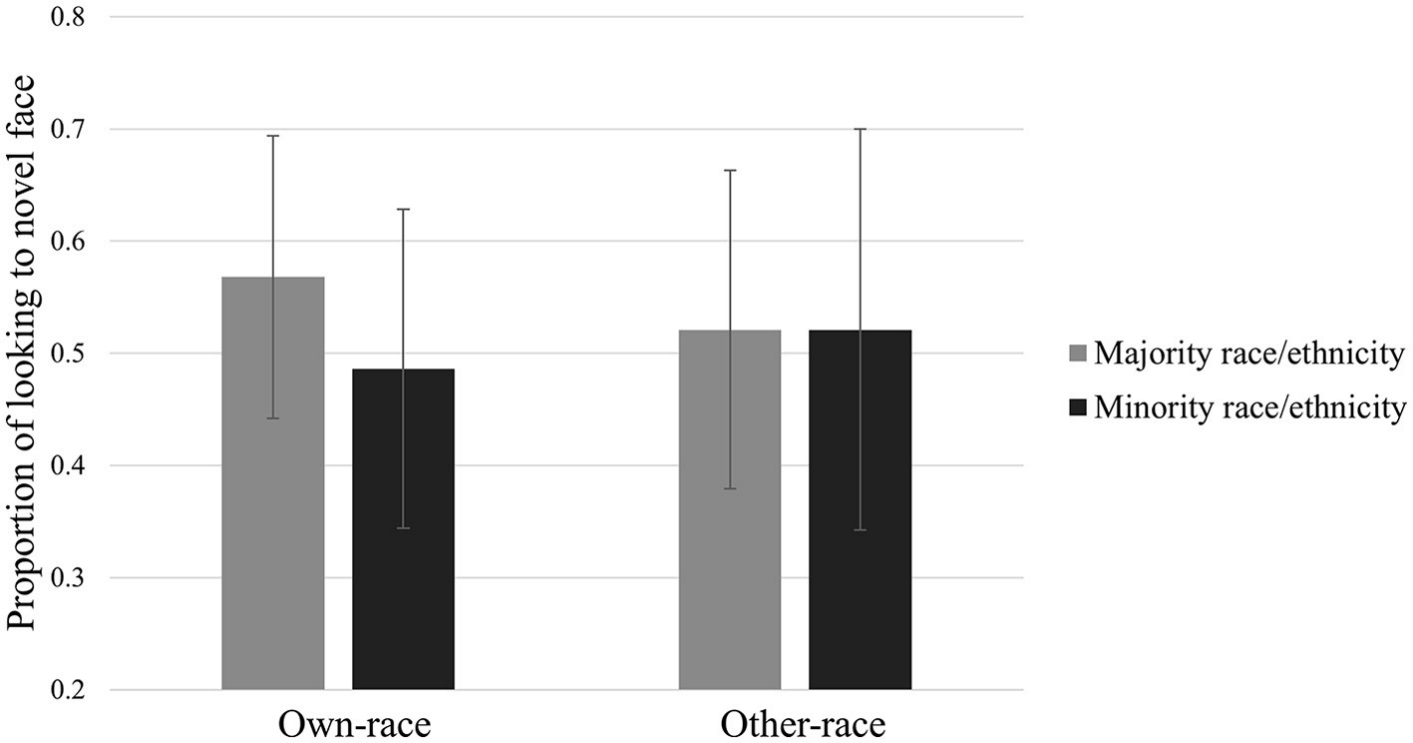
Proportion of looking at own- and other-race novel faces among demographic majority and minority members.

One-way ANOVAs were conducted to compare the effects of demographic majority status on the proportion of looking at own-race and other-race novel faces. There was a significant difference in the proportion of looking at own-race novel faces, *F*_(1,59)_ = 5.40, *p* = 0.024, $\eta_{p}^{2}$ = 0.084. Participants who had a racial or ethnic minority identity had significantly lower proportions of looking at novel own-race faces (*M* = 0.486, *SD* = 0.143) than participants who had a racial or ethnic majority identity (*M* = 0.568, *SD* = 0.126). There were no significant differences in the proportion of looking at other-race novel faces, *F*_(1,60)_ = < 0.001, *p* = 0.995, $\eta_{p}^{2}$ < 0.001. Participants who had a racial or ethnic minority identity did not differ in their proportions of looking at novel other-race faces (*M* = 0.521, *SD* = 0.141) compared with participants who had a racial or ethnic majority identity (*M* = 0.521, *SD* = 0.178). These results are presented in Figure 3.

### Community diversity and the other-race effect

Community diversity did not significantly predict the proportion of looking at own-race novel faces or the proportion of looking at other-race novel faces. In both the own- and other-race models including majority status as a dichotomous moderator, the diversity by minority status interaction was non-significant, indicating that there was no moderating effect of minority status on the relationship between diversity index score and proportion of looking at either own-race or other-race novel faces. The results of these regression models are presented in Table 3.

**Table 3:** Regression model results.

**Model**	***R* ^2^**	**Predictor**	**B**	**SE**	**β**	***t***	***p***
Diversity, minority status, and diversity^*^minority status predicting looking at novel own-race face	0.112	Constant	0.50	0.05		9.10	< 0.001
		Minority status	−0.09	0.04	−0.32	−2.42	0.019
		Diversity	0.15	0.11	0.20	1.29	0.204
		Minority status^*^diversity	−0.08	0.21	−0.06	−0.36	0.719
Diversity, minority status, and diversity^*^minority status predicting looking at novel other-race face	0.029	Constant	0.48	0.07		6.99	< 0.001
		Minority status	−0.02	0.05	−0.05	−0.40	0.691
		Diversity	0.09	0.15	0.09	0.58	0.563
		Minority status^*^diversity	0.18	0.26	−0.11	0.66	0.512

**Figure 4:**
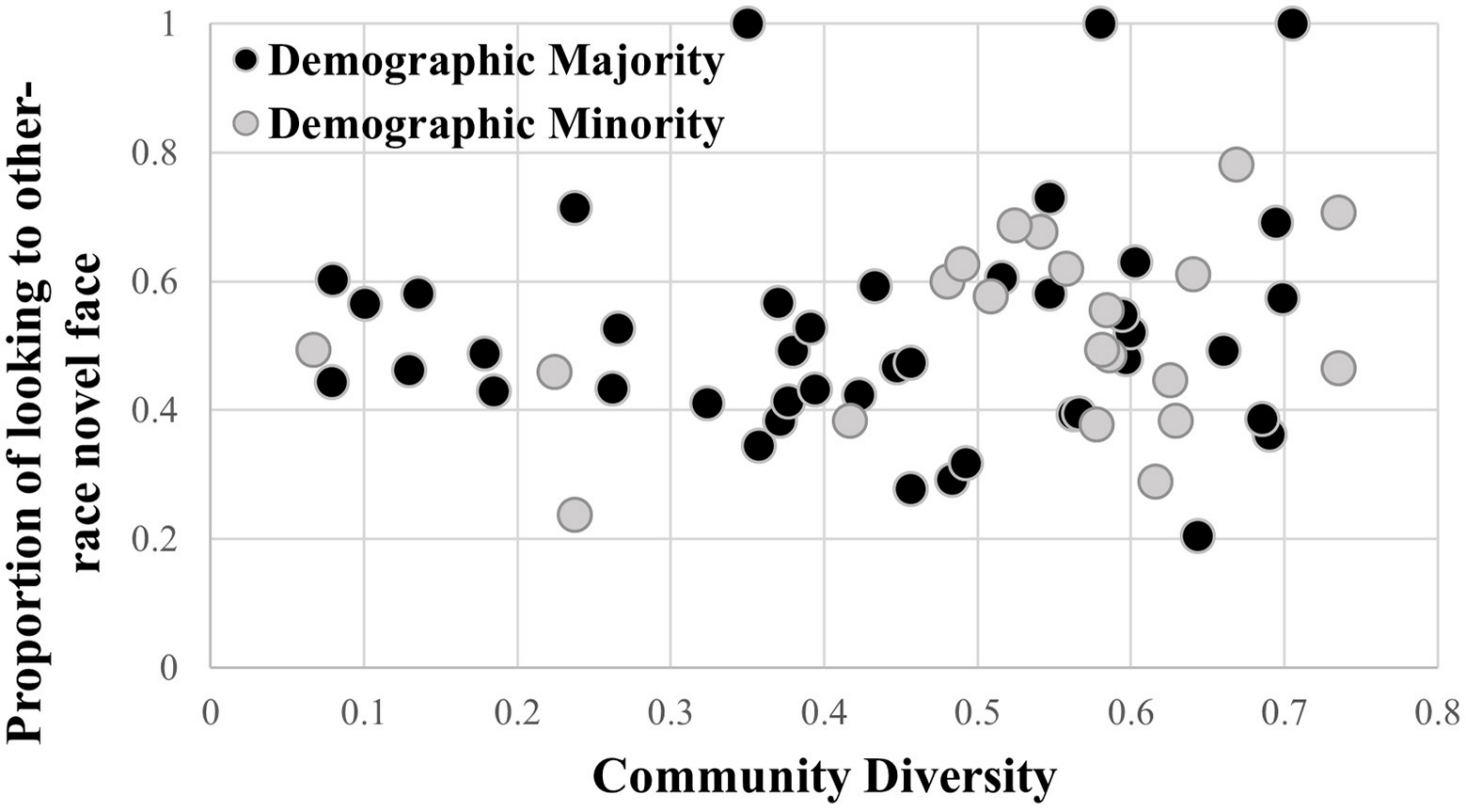
Community diversity by the proportion of looking at other-race novel face. Threshold for the recognition of the other-race face is indicated by the solid line.

Although the regression models showed non-significant results, a visual inspection of the relationship between community diversity and the proportion of looking at the other-race novel face revealed potential differences. This is presented in Figure 4. In particular, it appears that participants living in more diverse communities were more likely to demonstrate a preference for the other-race novel face than infants from communities with less diversity. The average HHI among infants who exhibited other-race face recognition was 0.53, compared with 0.44 in the subset of infants who did not exhibit other-race face recognition. These results must be interpreted with heavy caution, however, given that there is a wide range of novelty scores among infants from more diverse communities and the overall relationship is not statistically significant.

## Discussion

This study examined the role of community racial diversity on the development of the ORE in 9- to 12-month-old infants across the United States using a familiarization and VPC procedure administered asynchronously online. We hypothesized that infants who were exposed to greater levels of community diversity would exhibit the recognition of both own- and other-race faces, as opposed to infants from communities with lower levels of diversity, who were expected to demonstrate the other-race effect by exhibiting own-race recognition. We found evidence for the ORE in 9- to 12-month-olds, which was driven by the majority-race participants and weakened in the minority-race participants. However, we did not find a significant effect of the level of community racial diversity on the strength of the ORE.

The results of our study replicated prior research on the other-race effect; participants showed a novelty preference for own-race faces but not for other-race faces. These findings strengthen the current body of literature establishing ORE is developed and observable in 9- to 12-month-old infants, as evidenced by their ability to discriminate among faces of their own race but not faces of another race (Sangrigoli and De Schonen, 2004; Kelly et al., 2007, 2009; Quinn et al., 2018, 2019; Krasotkina et al., 2020; Pascalis et al., 2020). The current study also speaks to the robustness of the ORE by replicating prior findings using a racially heterogeneous sample and an online paradigm, both of which are fairly novel contributions to the ORE literature.

When considering participant racial and ethnic identity in our analyses, we found that infants who identified as a demographic minority group within their community demonstrated a weaker ORE. They did not demonstrate a novelty preference for either own- or other-race faces, indicating that the observed ORE in our overall findings was driven by infants belonging to a majority racial group. We posit that infants belonging to a minority group naturally have greater exposure to racial out-groups than infants belonging to a majority group and that this increased exposure mitigates the development of perceptual biases for own-race faces. These findings build upon previous studies on the ORE in infants belonging to a minority racial or ethnic group and on infants living in diverse communities (Sangrigoli et al., 2005; Bar-Haim et al., 2006; Sugden, 2016; Tham et al., 2019; Zhou et al., 2019, 2022; Hwang et al., 2021). This study is novel, however, in the diversity of the communities sampled. The data were representative of multiple racial and ethnic groups living in communities of varying levels of racial and ethnic diversity, which, to our knowledge, has only been investigated in two other studies that were both conducted outside of the United States.

When examining zip code level racial and ethnic diversity, our chosen measure of community diversity did not significantly predict the proportion of looking at either own- or other-race faces, indicating that community diversity did not significantly impact the strength of the ORE observed in our sample. Additionally, we did not find evidence that the relationship between community diversity and the ORE depended on whether participants belonged to a racial majority or minority group. Although the regression models showed non-significant results, a visual inspection of the relationship between community diversity and the proportion of looking at the other-race novel face revealed potential differences. Infants who exhibited a preference for the other-race novel face (proportion of looking at the other-race novel > 0.60) appeared to live in more highly diverse communities than infants who did not. However, a wide range of novelty scores were demonstrated among infants from more diverse communities. These results are exploratory and warrant further investigation.

This study provides nuance to prior studies indicating the strength of the ORE may depend on racially diverse experiences (Sangrigoli and De Schonen, 2004; Sangrigoli et al., 2005; Bar-Haim et al., 2006; Heron-Delaney et al., 2011; Spangler et al., 2013; Sugden, 2016; Tham et al., 2019; Zhou et al., 2019, 2022; Hwang et al., 2021). Specifically, our findings are important in the context of the emerging literature exploring whether community diversity may impact face processing and recognition. A recent study with adults has indicated that living in a multi-racial community may diminish the ORE (Zhou et al., 2022), while others find that small hometown size and low density may impair face recognition (Balas and Saville, 2017; Sunday et al., 2019). Furthermore, recent evidence has found that neighborhood diversity predicts the strength of the ORE in White infants at the neural level (Hwang et al., 2021). Taken together, the current body of evidence suggests that exposure to diversity has the potential to alter face recognition ability. The lack of statistical significance of diversity level in the current study may indicate that the impact of diverse face experience on the ORE is detectable at the neural level, but not at the behavioral level, at the ages sampled and may be more pronounced for face recognition skills in adulthood, but our findings are not without limitations and warrant future investigation. For example, our chosen measure of community diversity, the Hirschman–Herfindahl index calculated using the American Community Survey data, may not have adequately captured the nuance in infants' experiences with racial out-group members. Given that the community diversity measure was also uncorrelated with parent-reported exposure to individuals of other races, it is possible that our community-level measure of racial exposure did not reflect individual participants' experience with racial in-group and out-group members. However, a number of parents did not complete the parent-report measure, which may have impacted the results. Future studies would benefit from a more nuanced measure of individual exposure to racial and ethnic diversity that includes both community-level factors and individual self-report to capture infants' day-to-day experiences more accurately.

Furthermore, data collection for this study occurred in a unique time period after the height of the COVID-19 pandemic, which may contribute to our cohort of infants having less typical experiences with community socialization (Green et al., 2021; Carnevali et al., 2022). Research has suggested that infants born during and after the onset of the pandemic have had substantially fewer social interactions with people outside of their households (Kim et al., 2022). Neural and behavioral evidence have additionally supported the hypothesis that altered visual experience in infants resulting from the COVID-19 pandemic, including decreased socialization with others and exposure to masked faces, contributed to altered face recognition and processing abilities (Kim et al., 2022; Yates et al., 2023). Regardless, future research should aim to develop a more nuanced measure of exposure to racial out-groups that includes both quality and quantity of exposure in the home and community settings, as well as collect data on the racial and ethnic identities of the people the infant is regularly exposed to.

This study is among the first to test the ORE across more than one or two racial groups. Stimuli provided representation for those from Black, Asian, Hispanic/Latinx, and White backgrounds. However, race is a highly complex social construct that is not able to be captured with strictly categorical stimuli, so we were unable to match all participant's racial identities due to the limitations of the available stimulus categories. As the population of infants in the United States becomes increasingly diverse with greater representation of bi- and multi-racial identities, there is a growing need to create stimulus sets, procedures, and analyses that reflect greater inclusivity of racial and ethnic identity. Although our sample better reflected the racial and ethnic makeup of the United States than prior studies on the ORE, due to our limited sample size, we were unable to further break down our analyses to parse out any unique effects related to certain racial or ethnic identities. Future studies should aim to address this by recruiting larger samples as well as specifically recruiting individuals who identify with diverse racial or ethnic minority groups to examine whether the ORE operates differently among minoritized infants in the United States.

Participants completed the current study asynchronously utilizing the online platform Lookit (Scott and Schulz, 2017; Scott et al., 2017). Lookit has been used in a variety of recent infant studies conducted online (e.g., Nelson and Oakes, 2021; Luchkina and Waxman, 2022; Rocha and Addyman, 2022; Bursalioglu et al., 2023; Colomer and Woodward, 2023; Wang, 2023). The Lookit platform provided us the opportunity to obtain data from participants across the United States and allowed participants to flexibly complete the study on their own schedules. However, there were also limitations to this research approach. In particular, we had less control over the testing conditions than we would have had if a traditional lab-based setting had been used. Some participants were distracted by their home environments or may have had less visibility of the stimuli.

Overall, this study contributes to the current body of literature on the other-race effect and has important implications for the impact of environmental exposure to racial and ethnic diversity on the development of perceptual biases based on race. Our findings replicated the ORE using an online paradigm while also contributing more nuance to our understanding of the ORE in individuals who represent a racial or ethnic minority in their community within the United States. Attention to race in infancy has been described in a recent study as a possible “developmental entry point” for later racial biases, which may begin guiding children's behavior toward and expectations of others as early as the preschool age (Waxman, 2021). Our finding that infants of a majority racial group develop a stronger ORE than infants of a minority racial group is particularly important in the context of the United States as we seek to understand how implicit biases develop and how we may mitigate them. Because we posit that infants of a minority race may have more overall experiences with faces of another race, leading to a weakened or absent ORE, this study may have broader implications for social justice and diversity as positive influences on early development. Our evidence provides support for promoting diverse environments during infancy as a way to potentially mitigate early perceptual biases based on race in majority-race or ethnicity infants. The relative novelty of the study design and findings signals a need for a wider body of research exploring the relationships between experiences with racial and ethnic diversity and the development of the ORE in infancy and beyond.

## Data availability statement

The raw data supporting the conclusions of this article will be made available by the authors, without undue reservation.

## Ethics statement

The studies involving human participants were reviewed and approved by Loyola University Chicago Institutional Review Board. Written informed consent from the participants' legal guardian/next of kin was not required to participate in this study in accordance with the national legislation and the institutional requirements.

## Author contributions

TB and MG contributed to the conception and design of the study. TB and AB programmed the study. TB, CH, and AB processed the data. CH performed the statistical analysis. TB and CD created manuscript figures. TB and CH wrote the first draft of the manuscript. MG advised on statistical analyses. TB, CH, and MG created manuscript figures. All authors contributed to manuscript revision, read, and approved the submitted version.

## References

[B1] Awaworyi Churchill S. Farrell L. Smyth R. (2019). Neighbourhood ethnic diversity and mental health in Australia. Health Econ. 28, 1075–1087. 10.1002/hec.3928 31290216

[B2] Balas B. Saville A. (2017). Hometown size affects the processing of naturalistic face variability. Vision Res. 141, 228–236. 10.1016/j.visres.2016.12.005 28025050 PMC5494272

[B3] Bar-Haim Y. Ziv T. Lamy D. Hodes R. M. (2006). Nature and nurture in own-race face processing. Psychol. Sci. 17, 159–163. 10.1111/j.1467-9280.2006.01679.x 16466424

[B4] Bayet L. (2022). How infants learn from a world of faces: implications for racial biases and mask-wearing. Policy Insights Behav. Brain Sci. 9, 65–72. 10.1177/23727322211068007

[B5] Burns E. J. Tree J. Chan A. H. Xu H. (2019). Bilingualism shapes the other race effect. Vision Res. 157, 192–201. 10.1016/j.visres.2018.07.004 30102922

[B6] Bursalioglu A. Michalak A. Guy M. W. (2023). Intersensory redundancy impedes face recognition in 12-month-old infants. Front. Psychol. 14, 1210132. 10.3389/fpsyg.2023.1210132 37529309 PMC10389088

[B7] Carnevali L. Gui A. Jones E. J. Farroni T. (2022). Face processing in early development: a systematic review of behavioral studies and considerations in times of COVID-19 pandemic. Front. Psychol. 388, 778247. 10.3389/fpsyg.2022.778247 35250718 PMC8894249

[B8] Clerc O. Fort M. Schwarzer G. Krasotkina A. Vilain A. Méary D.. (2022). Can language modulate perceptual narrowing for faces? Other-race face recognition in infants is modulated by language experience. Int. J. Behav. Dev. 46, 83–90. 10.1177/01650254211053054

[B9] Colomer M. Woodward A. (2023). Should I learn from you? Seeing expectancy violations about action efficiency hinders social learning in infancy. Cognition 230, 105293. 10.1016/j.cognition.2022.105293 36191356

[B10] Conley M. I. Dellarco D. V. Rubien-Thomas E. Cohen A. O. Cervera A. Tottenham N.. (2018). The racially diverse affective expression (RADIATE) face stimulus set. Psychiatry Res. 270, 1059–1067. 10.1016/j.psychres.2018.04.066 29910020 PMC6446554

[B11] Damon F. Quinn P. C. Méary D. Pascalis O. (2022). Asymmetrical responding to male versus female other-race categories in 9-to 12-month-old infants. Br. J. Psychol. 114(Suppl. 1):71–93. 10.1111/bjop.12582 35808935

[B12] Datavyu Team (2014). Datavyu: A Video Coding Tool. Databrary Project. New York University. Available online at: http://datavyu.org (accessed April 26, 2023).

[B13] Fagan J. F. (1970). Memory in the infant. J. Exp. Child Psychol. 9, 217–226. 10.1016/0022-0965(70)90087-1 5452116

[B14] Fantz R. L. (1956). A method for studying early visual development. Percept. Mot. Skill 6, 13–15. 10.2466/pms.1956.6.g.13

[B15] Fassbender I. Teubert M. Lohaus A. (2016). The development of preferences for own-race versus other-race faces in 3-, 6-and 9-month-old Caucasian infants. Eur. J. Dev. Psychol. 13, 152–165. 10.1080/17405629.2015.107358525664830

[B16] Goren C. C. Sarty M. Wu P. Y. (1975). Visual following and pattern discrimination of face-like stimuli by newborn infants. Pediatrics 56, 544–549. 10.1542/peds.56.4.544 1165958

[B17] Green J. Staff L. Bromley P. Jones L. Petty J. (2021). The implications of face masks for babies and families during the COVID-19 pandemic: a discussion paper. J. Neonatal Nurs. 27, 21–25. 10.1016/j.jnn.2020.10.005 33162776 PMC7598570

[B18] Heron-Delaney M. Anzures G. Herbert J. S. Quinn P. C. Slater A. M. Tanaka J. W.. (2011). Perceptual training prevents the emergence of the other race effect during infancy. PLoS ONE 6, e19858. 10.1371/journal.pone.0019858 21625638 PMC3097220

[B19] Hillairet de Boisferon A. Kubicek C. Gervain J. Schwarzer G. Loevenbruck H. Vilain A.. (2021). Language familiarity influences own-race face recognition in 9-and 12-month-old infants. Infancy 26, 647–659. 10.1111/infa.12404 33988894

[B20] Hirschman A. O. (1964). The paternity of an index. Am. Econ. Rev. 54, 761.

[B21] Hwang H. G. Debnath R. Meyer M. Salo V. C. Fox N. A. Woodward A. (2021). Neighborhood racial demographics predict infants' neural responses to people of different races. Dev. Sci. 24, e13070. 10.1111/desc.13070 33277794 PMC8522049

[B22] Johnson M. H. Dziurawiec S. Ellis H. Morton J. (1991). Newborns' preferential tracking of face-like stimuli and its subsequent decline. Cognition 40, 1–19. 10.1016/0010-0277(91)90045-6 1786670

[B23] Kelly D. J. Liu S. Lee K. Quinn P. C. Pascalis O. Slater A. M.. (2009). Development of the other-race effect during infancy: evidence toward universality?. J. Exp. Child Psychol. 104, 105–114. 10.1016/j.jecp.2009.01.006 19269649 PMC3740564

[B24] Kelly D. J. Quinn P. C. Slater A. M. Lee K. Ge L. Pascalis O. (2007). The other-race effect develops during infancy: evidence of perceptual narrowing. Psychol. Sci. 18, 1084–1089. 10.1111/j.1467-9280.2007.02029.x 18031416 PMC2566514

[B25] Kelly D. J. Quinn P. C. Slater A. M. Lee K. Gibson A. Smith M.. (2005). Three-month-olds, but not newborns, prefer own-race faces. Dev. Sci. 8, F31–F36. 10.1111/j.1467-7687.2005.0434a.x 16246233 PMC2566511

[B26] Kim K. K. Fang W. Liu A. Panesar D. Xiao N. G. (2022). Altered development of face recognition among infants born amid the COVID-19 pandemic. PsyArXiv [Preprint]. 10.31234/osf.io/n96fv 38703752

[B27] Krasotkina A. Götz A. Höhle B. Schwarzer G. (2020). Infants' gaze patterns for same-race and other-race faces, and the other-race effect. Brain Sci. 10, 331. 10.3390/brainsci10060331 32486016 PMC7349221

[B28] Lai C. K. Skinner A. L. Cooley E. Murrar S. Brauer M. Devos T.. (2016). Reducing implicit racial preferences: II. Intervention effectiveness across time. J. Exp. Psychol. Gen. 145, 1001. 10.1037/xge0000179 27454041

[B29] Lee K. Quinn P. C. Pascalis O. (2017). Face race processing and racial bias in early development: a perceptual-social linkage. Curr. Dir. Psychol. Sci. 26, 256–262. 10.1177/0963721417690276 28751824 PMC5523824

[B30] Liu S. Xiao W. S. Xiao N. G. Quinn P. C. Zhang Y. Chen H.. (2015). Development of visual preference for own-versus other-race faces in infancy. Dev. Psychol. 51, 500. 10.1037/a0038835 25664830

[B31] Luchkina E. Waxman S. (2022). “Semantic priming supports infants' ability to represent and name unseen objects,” in Proceedings of the Annual Meeting of the Cognitive Science Society. p. 44. Available online at: https://escholarship.org/uc/item/8jw3q79r

[B32] Macchi Cassia V. Bulf H. Quadrelli E. Proietti V. (2014). Age-related face processing bias in infancy: evidence of perceptual narrowing for adult faces. Dev. Psychobiol. 56, 238–248. 10.1002/dev.21191 24374735

[B33] Markant J. Scott L. S. (2018). Attention and perceptual learning interact in the development of the other-race effect. Curr. Dir. Psychol. Sci. 27, 163–169. 10.1177/096372141876988434427494

[B34] Marquis A. R. Sugden N. A. (2019). Meta-analytic review of infants' preferential attention to familiar and unfamiliar face types based on gender and race. Dev. Rev. 53, 100868. 10.1016/j.dr.2019.100868

[B35] Nelson C. M. Oakes L. M. (2021). “May I Grab Your Attention?”: an investigation into infants' visual preferences for handled objects using Lookit as an online platform for data collection. Front. Psychol. 12, 733218. 10.3389/fpsyg.2021.733218 34566820 PMC8460868

[B36] Nosek B. A. Smyth F. L. Hansen J. J. Devos T. Lindner N. M. Ranganath K. A.. (2007). Pervasiveness and correlates of implicit attitudes and stereotypes. Eur. Rev. Soc. Psychol. 18, 36–88. 10.1080/1046328070148905312972672

[B37] Pascalis O. Fort M. Quinn P. C. (2020). Development of face processing: are there critical or sensitive periods? Curr. Opin. Behav. Sci. 36, 7–12. 10.1016/j.cobeha.2020.05.005

[B38] Quinn P. C. Lee K. Pascalis O. (2018). Perception of face race by infants: five developmental changes. Child Dev. Perspect. 12, 204–209. 10.1111/cdep.12286

[B39] Quinn P. C. Lee K. Pascalis O. (2019). Face processing in infancy and beyond: the case of social categories. Annu. Rev. Psychol. 70, 165–189. 10.1146/annurev-psych-010418-102753 30609912

[B40] Quinn P. C. Lee K. Pascalis O. (2020). Beyond perceptual development: infant responding to social categories. Adv. Child Dev. 58, 35–61. 10.1016/bs.acdb.2020.01.002 32169198

[B41] Quinn P. C. Lee K. Pascalis O. Tanaka J. W. (2016). Narrowing in categorical responding to other-race face classes by infants. Dev. Sci. 19, 362–371. 10.1111/desc.12301 25899938

[B42] Quinn P. C. Uttley L. Lee K. Gibson A. Smith M. Slater A. M.. (2008). Infant preference for female faces occurs for same-but not other-race faces. J. Neuropsychol. 2, 15–26. 10.1348/174866407X231029 19334302

[B43] Quinn P. C. Yahr J. Kuhn A. Slater A. M. Pascalis O. (2002). Representation of the gender of human faces by infants: a preference for female. Perception 31, 1109–1121. 10.1068/p3331 12375875

[B44] Rhoades S. A. (1993). The herfindahl-hirschman index. Fed. Res. Bull. 79, 188.

[B45] Rhodes M. Baron A. (2019). The development of social categorization. Annu. Rev. Dev. Psychol. 1, 359. 10.1146/annurev-devpsych-121318-084824 33103119 PMC7577394

[B46] Richmond J. Colombo M. Hayne H. (2007). Interpreting visual preferences in the visual paired-comparison task. J. Exp. Psychol. Learn. Mem. Cogn. 33, 823. 10.1037/0278-7393.33.5.823 17723062

[B47] Rocha S. Addyman C. (2022). Assessing sensorimotor synchronisation in toddlers using the Lookit online experiment platform and automated movement extraction. Front. Psychol. 13, 897230. 10.3389/fpsyg.2022.897230 35846621 PMC9282044

[B48] Sangrigoli S. De Schonen S. (2004). Recognition of own-race and other-race faces by three-month-old infants. JCPP 45, 1219–1227. 10.1111/j.1469-7610.2004.00319.x 15335342

[B49] Sangrigoli S. Pallier C. Argenti A. M. Ventureyra V. A. G. de Schonen S. (2005). Reversibility of the other-race effect in face recognition during childhood. Psychol. Sci. 16, 440–444. 10.1111/j.0956-7976.2005.01554.x 15943669

[B50] Scott K. Chu J. Schulz L. (2017). Lookit (Part 2): assessing the viability of online developmental research, results from three case studies. Open Mind 1, 15–29. 10.1162/OPMI_a_00001

[B51] Scott K. Schulz L. (2017). Lookit (part 1): a new online platform for developmental research. Open Mind 1, 4–14. 10.1162/OPMI_a_00002

[B52] Scott L. S. Pascalis O. Nelson C. A. (2007). A domain-general theory of the development of perceptual discrimination. Curr. Direct. Psychol. Sci. 16, 197–201. 10.1111/j.1467-8721.2007.00503.x 21132090 PMC2995946

[B53] Serafini L. Pesciarelli F. (2022). Neural timing of the other-race effect across the lifespan: a review. Psychophysiology 60, e14203. 10.1111/psyp.14203 36371686

[B54] Spangler S. M. Schwarzer G. Freitag C. Vierhaus M. Teubert M. Fassbender I.. (2013). The other-race effect in a longitudinal sample of 3-, 6-and 9-month-old infants: evidence of a training effect. Infancy 18, 516–533. 10.1111/j.1532-7078.2012.00137.x

[B55] Sturgis P. Brunton-Smith I. Kuha J. Jackson J. (2014). Ethnic diversity, segregation and the social cohesion of neighbourhoods in London. Ethn. Racial Stud. 37, 1286–1309. 10.1080/01419870.2013.83193228989199

[B56] Sugden N. A. (2016). Learning from Experience: Exposure to, Attention to, Discrimination of, and Brain Response to Faces at 3, 6, and 9 Months (Doctoral dissertation). Ryerson University.

[B57] Sugden N. A. Marquis A. R. (2017). Meta-analytic review of the development of face discrimination in infancy: Face race, face gender, infant age, and methodology moderate face discrimination. Psychol. Bull. 143, 1201. 10.1037/bul0000116.supp 28758764

[B58] Sugden N. A. Moulson M. C. (2019). These are the people in your neighbourhood: consistency and persistence in infants' exposure to caregivers', relatives', and strangers' faces across contexts. Vision Res. 157, 230–241. 10.1016/j.visres.2018.09.005 30291919

[B59] Sunday M. A. Patel P. A. Dodd M. D. Gauthier I. (2019). Gender and hometown population density interact to predict face recognition ability. Vision Res. 163, 14–23. 10.1016/j.visres.2019.08.006 31472340

[B60] Tham D. S. Y. Woo P. J. Bremner J. G. (2019). Development of the other-race effect in Malaysian-Chinese infants. Dev. Psychobiol. 61, 107–115. 10.1002/dev.21783 30239984

[B68] Tottenham N. Tanaka J. W. Leon A. C. McCarry T. Nurse M. Hare T. A.. (2009). The NimStim set of facial expressions: Judgments from untrained research participants. *Psychiatry Res*. 168, 242–249. 10.1016/j.psychres.2008.05.006 19564050 PMC3474329

[B61] U.S. Census Bureau (2022). American Community Survey Data. Available online at: https://www.census.gov/programs-surveys/acs (accessed April 26, 2023).

[B62] Ujiie Y. Kanazawa S. Yamaguchi M. K. (2021). The other-race effect on the McGurk effect in infancy. Atten. Percept. Psychophys. 83, 2924–2936. 10.3758/s13414-021-02342-w 34386882 PMC8460584

[B63] Wang J. (2023). Does virtual counting count for babies? Evidence from an online looking time study. Develop. Psychol. 59, 669–675. 10.1037/dev0001478 36342439

[B64] Waxman S. R. (2021). Racial awareness and bias begin early: developmental entry points, challenges, and a call to action. Perspect. Psychol. Sci. 16, 893–902. 10.1177/17456916211026968 34498529

[B65] Yates T. S. Ellis C. T. Turk-Browne N. B. (2023). Face processing in the infant brain after pandemic lockdown. Dev. Psychobiol. 65, e22346. 10.1002/dev.22346 36567649 PMC9877889

[B66] Zhou X. Elshiekh A. Moulson M. C. (2019). Lifetime perceptual experience shapes face memory for own-and other-race faces. Vis. Cogn. 27, 687–700. 10.1080/13506285.2019.1638478

[B67] Zhou X. Mondloch C. J. Chien S. H. L. Moulson M. C. (2022). Multi-cultural cities reduce disadvantages in recognizing naturalistic images of other-race faces: evidence from a novel face learning task. Sci. Rep. 12, 8950. 10.1038/s41598-022-11550-9 35624118 PMC9142532

